# Disparities in disease profiles among Afro-Brazilians and public healthcare patients with prostate cancer: real-world data from Brazil

**DOI:** 10.31744/einstein_journal/2026AO2267

**Published:** 2026-08-12

**Authors:** Ana Luiza Spina Nagy, Yuri Greb Vazquez, Yasmin Giovani de Sousa, Natalia Yamazaki Centrone, Uelson Donizeti Rocioli, Francisco Tustumi, Fernando Moura, Fernando Maluf, Pedro Luiz Serrano Usón

**Affiliations:** 1 Hospital Israelita Albert Einstein Faculdade Israelita de Ciências da Saúde Albert Einstein São Paulo SP Brazil Faculdade Israelita de Ciências da Saúde Albert Einstein, Hospital Israelita Albert Einstein, São Paulo, SP, Brazil.; 2 Hospital Israelita Albert Einstein Center for Personalized Medicine São Paulo SP Brazil Center for Personalized Medicine, Hospital Israelita Albert Einstein, São Paulo, SP, Brazil.; 3 Hospital Israelita Albert Einstein Hospital Municipal Dr. Gilson de Cássia Marques de Carvalho São Paulo SP Brazil Hospital Municipal Dr. Gilson de Cássia Marques de Carvalho; Hospital Israelita Albert Einstein, São Paulo, SP, Brazil.

**Keywords:** Prostatic neoplasms, Equity, Public policy, Ethics, Inequality, Health policy

## Abstract

**Objective:**

To evaluate disparities in patients with prostate cancer treated at private or public health systems in Brazil.

**Methods:**

Anonymized retrospective and prospective data were collected from the medical records of patients over 18 years old diagnosed with prostate cancer between 2017 and 2024.

**Results:**

A descriptive analysis of 2,387 patients treated at private or public hospitals revealed that more than half were younger than 75 years old. Regarding race, 58% of patients reported their ethnicity: 75.41% identified as white, 13.84% mixed-race, 7.35% Black, 3.32% Asian, and 0.07% Indigenous. Public hospital patients had a 2.5-fold higher risk of metastasis at diagnosis (p<0.01) and longer hospital stays (p<0.01) when compared to private service patients. Black (odds ratio [OR]=2.27) and mixed-race (OR=1.89) patients had a higher risk of metastasis at diagnosis than white patients. Furthermore, Black (OR=0.04), mixed-race (OR=0.26), and Asian (OR=0.14) patients, and those >75 years old (OR=0.42) had lower chances of undergoing advanced surgery techniques such as robotic surgery. Patients older than 75 years had significantly worse overall survival (p<0.01).

**Conclusion:**

The results of this study highlight significant disparities in patients with prostate cancer. Black and mixed-race patients, as well as those from the public healthcare system, faced a higher risk of metastasis at diagnosis and lower chances of accessing advanced technologies. Additionally, advanced age was associated with shorter overall survival and longer hospitalization. These findings underscore the need for targeted interventions to reduce treatment access inequalities and improve clinical outcomes, particularly in vulnerable populations.

## Highlights

Descriptive analysis of 2,387 patients treated at private and public hospitals.

Public patients had a 2.5 fold higher risk of metastasis at diagnosis when compared to private service patients, and longer hospital stays.

Regarding race, Black and mixed-race patients had a higher risk of metastasis at diagnosis than whites and a lower chance of undergoing advanced surgery techniques such as robotic surgery.

Patients aged more than 75 years have statistically worse overall survival.

## In Brief

In this study, we sought to evaluate disparities in prostate cancer treatment between private and public hospitals. Black and mixed-race patients, as well as those treated in the public system, faced a higher risk of metastasis at diagnosis and lower chances of accessing advanced technologies. These findings underscore the need for targeted interventions to reduce treatment access inequalities.

## Graphical abstract

**Figure:**
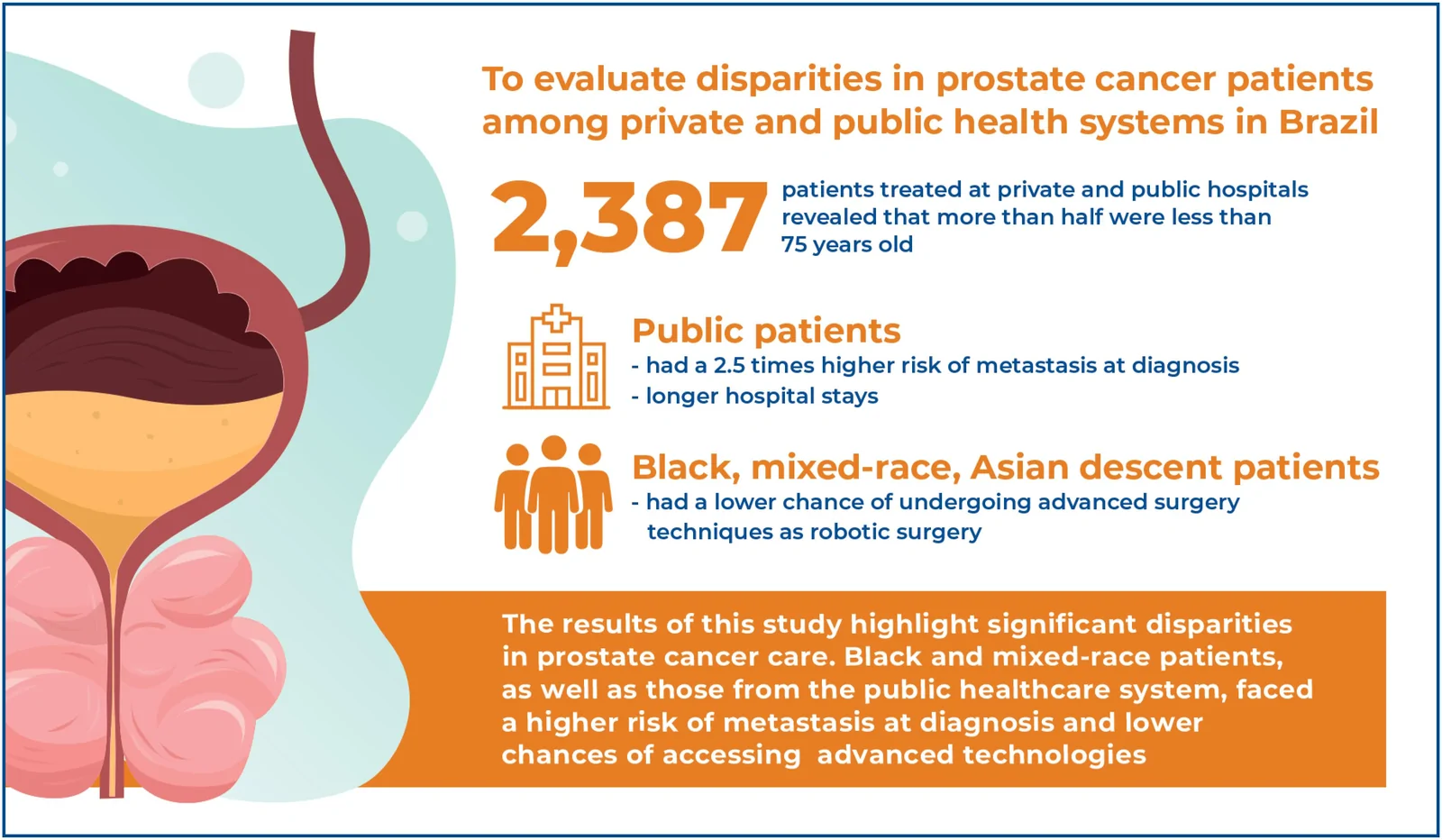

## INTRODUCTION

Prostate cancer is the most common malignancy among men worldwide and the second leading cause of cancer-related death in men, disproportionately affecting Afro-descendant populations.^(1)^ Globally, over 1.4 million new cases of prostate cancer are diagnosed annually, with notable disparities in incidence and outcomes based on geographic, racial, and socioeconomic factors.^(1)^ Brazil mirrors this trend, with approximately 70,000 new prostate cancer cases diagnosed each year.^(2)^ Afro-descendant patients, in particular, experience more aggressive forms of the disease and worse survival rates compared to white patients.^(3)^ These disparities are exacerbated by socioeconomic barriers that limit access to timely diagnosis and treatment, particularly in public healthcare systems.^(4)^

Studies conducted in the United States and Europe have shown that Black men are more likely to develop aggressive prostate cancer and are often diagnosed at later stages. Research also highlights that Black men face worse treatment outcomes and higher mortality rates than white men.^(5)^ This pattern is echoed in Brazil, where structural barriers in the public healthcare system delay diagnoses and result in suboptimal treatment, particularly for Afro-descendant populations.^(4,6)^

Socioeconomic status further contributes to disparities in prostate cancer outcomes. Access to advanced diagnostic tools and treatments, such as prostate-specific antigen screening and robotic surgery, is often limited in public hospitals, exacerbating health inequities between different racial and socioeconomic groups.^(7)^

This study aimed to explore these disparities by comparing prostate cancer outcomes between Black and white patients treated in public and private healthcare settings in Brazil. The study focused on the outcomes of patients treated at *Hospital Israelita Albert Einstein* (HIAE), a leading private institution in South America, or at *Hospital Municipal Vila Santa Catarina* (HMVSC), a public hospital with a high patient volume.

## OBJECTIVE

To evaluate disparities in patients with prostate cancer treated at private and public health systems in Brazil.

## METHODS

### Ethics statement

This study was conducted in accordance with the Declaration of Helsinki (2013 revision). All experimental protocols were approved based on Brazilian national standards. The study was approved by the ethics committee of *Hospital Israelita Albert Einstein* (CAAE: 83683824.7.0000.0071; # 7.208.109). Informed consent was waived due to the retrospective nature of the study and its low risk. Data were accessed for research purposes and analysis on 06/05/2025.

### Patients

Patients aged 18 years and older with prostate cancer treated at HIAE or HMVSC between January 2017 and December 2024 were retrospectively evaluated. Data collection included patient demographics (sex, race, age, diagnosis date, ZIP code, and distance from the treatment center) as well as disease characteristics and treatment types. Pathological data, staging (metastatic versus non-metastatic), and treatments (immunotherapy, hormone therapy, chemotherapy, targeted therapy, and robotic surgery) were recorded. Overall survival (OS) was calculated from the date of diagnosis to the date of death or last follow-up. Data from the hospital's Big Data/Analytics system program is available on request.

### Statistical analysis

OS was estimated using the Kaplan-Meier method. Cox regression, linear, and logistic models were applied to identify significant variables. Statistical significance for OS was set at p<0.05, and the results are presented as odds ratios (ORs). For all other tests, statistical significance was set at p<0.01. All analyses were performed using STATA software (version 16.1; Stata Corp LLC, College Station, TX, USA).

## RESULTS

A total of 2,387 patients aged 18 years or older diagnosed with prostate cancer were included in this study, with treatments administered at both systems between 2017 and 2024. In the cohort, 2,077 patients (87.1%) were younger than 75 years old, while 309 (12.95%) were older than 75. Racial demographics were reported by 58% of the patients, with 1,046 (75.41%) identifying as white, 192 (13.84%) as mixed-race, 102 (7.35%) as Black, 46 (3.32%) as Asian, and 1 (0.07%) as Indigenous.

Most patients (72.51%) were treated at private services, with the remaining 27.49% treated at public services. Distance to the treatment center was also a significant factor, as 61.48% of patients resided more than 10km from their respective treatment centers (Table 1).

**Table 1:** Patient clinical characteristics

n		Total 2,387		Private care		Public care		p value
				1,730	72.5%	656	27.5%	
		Count or mean	% or SD	Count or mean	% or SD	Count or mean	% or SD	
Age								
	≤75 years old	2,077	87.1	1495	86.4	582	88.7	0.135
	>75 years old	309	12.9	235	13.6	74	11.3	
Age								
	Mean±SD	65,7	8.8	65.6	9.1	65.9	7.9	0.448
Race								
	White	1,046	75.4	678	39.2	368	56,1	<0.001
	Mixed	192	13.8	37	2.1	155	23.6	
	Black	102	7.3	7	<0.1	95	14.5	
	Asian	46	3.3	9	<0.1	37	5.6	
	Indigenous	1	<0.1	0	0	1	<0.1	
Marital status								
	Married	173	86.5	167	86.5	6	85.7	0.951
	Single or divorced	27	13.5	26	13.5	1	14.3	
Cancer stage								
	Metastasis at diagnosis	491	20.7	277	16	214	67.1	<0.001
Distance from hospital								
	≤10km	867	38.5	651	37.6	216	33	0.001
	>10km	1384	61.5	946	54.7	438	67	
Length of hospital stay (days)								
Mean±SD		2,7	3	59.6	52.7	90.3	128.5	<0.001
Genetic test performed		80	3.4	79	4.6	1	<0.1	<0.001
Treatment								
	Targeted therapy	9	<0.1	9	0.5	0	0	0.064
	Immunotherapy	17	<0.1	17	1	0	0	0.011
	Hormone therapy	703	29.5	285	16.5	418	63.7	<0.001
	Chemotherapy	129	5.4	65	3.8	64	9.8	<0.001
	Surgery	1799	75.4	1470	85	329	19	<0.001
	Robotic surgery	1147	81.7	1147	66.3	0	0	<0.001
	Prostatectomy	1468	61.5	1264	73.1	204	11.8	<0.001
	Orchiectomy	13	<0.1	7	<0.1	6	<0.1	0.131
	Endoscopic resection	135	5.7	88	5.1	4	<0.1	<0.001

SD: standard deviation.

### Metastasis at diagnosis

After adjusting for race, location, and distance from the treatment center, patients treated under the Brazilian Public Health System (SUS - *Sistema Único de Saúde*) were found to have a 2.56-fold greater likelihood of being diagnosed with metastatic prostate cancer compared to those treated at HIAE (OR=2.56, 95% confidence interval [95%CI]=2.08-3.15, p<0.01). Racial differences were also significant. Black patients had an OR=2.27 (95%CI=1.62-3.20, p<0.01), and mixed-race patients had an OR=1.89 (95%CI=1.45-2.46, p<0.01) for metastatic disease at diagnosis compared to white patients (Table 2).

**Table 2:** Univariate and multivariate analysis of clinical characteristics

Metastatic disease at diagnosis		Univariate				Multivariate			
		OR	p value	95% CI		OR	p value	95% CI	
				LL	UL			LL	UL
Hospital	HIAE	ref.	-	-		ref.			
	HMVSC	2.56	<0.01	2.08	3.15	2.85	<0.01	2.13	3.82
Marital status	Married	ref.	-	-	-	ref.	-	-	-
	Single/Divorced	1.48	0.47	0.51	4.30	1.48	0.47	0.51	4.30
Race	White	ref.				ref.			
	Black	2.27	<0.01	1.48	3.50	1.40	0.15	0.89	2.21
	Asian	1.45	0.28	0.74	2.86	0.91	0.80	0.45	1.83
	Indigenous	1.00	-	-	-	1.00	-	-	-
Age	≤75 years	ref.				ref.			
	>75 years	1.83	<0.01	1.40	2.39	1.98	<0.01	1.40	2.81
Hospital distance	≤10 km	ref.				ref.			
	>10 km	0.92	0.45	0.75	1.14	0.92	0.45	0.75	1.14

95%CI: 95% confidence interval; HIAE: *Hospital Israelita Albert Einstein*; HMVSC: *Hospital Municipal Vila Santa Catarina*; LL: lower limit; OR: odds ratio; UL: upper limit.

### Access to robotic surgery

In terms of treatment modality, multivariate analysis revealed that Black patients had 96% reduced likelihood of undergoing robotic surgery compared to white patients (OR=0.04, 95%CI=0.02-0.09, p<0.01). Similarly, mixed-race and Asian patients had 74% (OR=0.26, 95%CI=0.18-0.38, p<0.01) and 86% (OR=0.14, 95%CI=0.07-0.29, p<0.01), respectively, lower likelihoods of receiving robotic surgery. Additionally, patients older than 75 years (OR=0.42, 95%CI=0.31-0.57, p<0.01) and those diagnosed with metastatic prostate cancer (OR=0.37, 95%CI=0.26-0.52, p<0.01) were less likely to receive robotic surgery. Distance was also a significant factor, with patients living more than 10 km from the treatment center being 95% less likely to undergo robotic surgery (Table 3).

**Table 3:** Univariate and multivariate analysis of advanced surgery and clinical characteristics

Robotic Surgery		Univariate				Multivariate			
		OR	p value	95% CI		OR	p value	95% CI	
				LL	UL			LL	UL
Race	White	ref.				ref.			
	Mixed	0.28	<0.01	0.16	0.49	0.26	<0.01	0.15	0.48
	Black	0.04	<0.01	0.01	0.14	0.04	<0.01	0.01	0.13
	Indigenous	1.00	-	-	-	1.00	-	-	-
Age	≤75 years	ref.				ref.			
	>75 years	0.37	<0.01	0.25	0.55	0.42	<0.01	0.25	0.71
Hospital distance	≤10km	ref.							
	>10km	1.29	0.07	0.98	1.71				

95#CI: 95% confidence interval; LL: lower limit; OR: odds ratio; UL: upper limit.

### Hospital stay duration

Patients treated at HMVSC (OR=28.87, 95%CI= 21.30-39.12, p<0.01), those aged over 75 years (OR=30.49, 95%CI=22.51-41.30, p<0.01), and those diagnosed with metastatic disease (OR=25.30, 95%CI=18.67-34.30, p<0.01) had significantly longer hospital stays. Patients in the public healthcare system had a cumulative average hospital stay of 90.3 days, while those in the private system stayed an average of 59.6 days. Despite this disparity, there is a noticeable congruence in data variability, as both systems exhibited high standard deviations (128.5 in the private system and 52.7 in the public system).

### Overall survival

Median OS was significantly shorter in patients over 75 years old and those diagnosed with metastatic disease than for younger patients and those without metastases (p<0.05; Figure 1).

**Figure 1:**
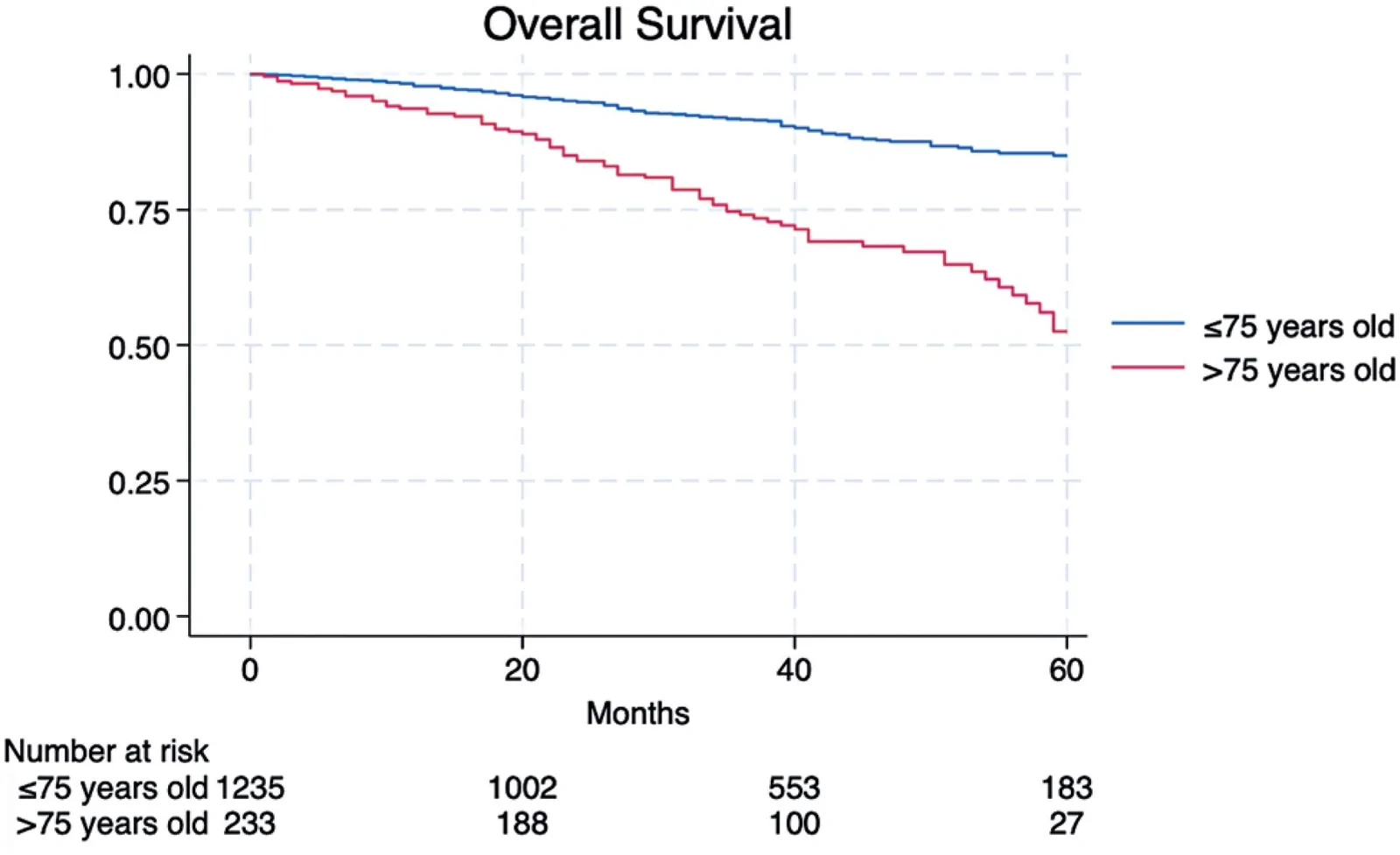
Overall survival of patients older or younger than 75 years old

## DISCUSSION

Racial disparities play a central role in the unequal outcomes for patients with prostate cancer in Brazil. Black patients were significantly more likely to be diagnosed with metastatic disease compared to white patients, highlighting broader socioeconomic inequalities and healthcare access challenges. These disparities were especially pronounced in public hospitals, where Black patients often presented with advanced disease, resulting in poor prognoses and intensive treatment requirements. This finding aligns with other studies showing that inequalities in healthcare access led to delayed diagnoses, poor outcomes for minority groups, and are likely associated with higher costs.^(8-10)^

An additional inequality was the impact of distance from treatment centers. Patients living more than 10km away from the hospital were 95% less likely to undergo robotic surgery, indicating that geographic barriers also play a role in access to advanced treatments. This is a crucial aspect that amplifies the disparities already caused by racial and socioeconomic factors.

The higher incidence of metastatic prostate cancer among Black patients directly correlated with long hospital stays and high treatment costs. Patients with metastatic disease require complex interventions, which may not be readily available in public hospitals, increasing the strain on the healthcare system. Prolonged hospitalizations not only elevate costs but also limit the availability of beds for other patients, exacerbating the burden on the healthcare system.

In addition, age and metastatic disease were significant factors for treatment and survival outcomes. Patients over 75 years old, as well as those diagnosed with metastasis, were less likely to receive robotic surgery and had poor survival rates. These findings reinforce the need to address both age-related and advanced disease challenges in the context of access to specialized treatments and are aligned with data from Brazilian patients published previously.^(9-11)^

In contrast, patients treated at private hospitals benefit from early diagnoses and access to advanced treatments, such as robotic surgery, targeted therapy, and immunotherapy; none of which were available in the SUS, as shown in Table 1. These advanced treatments contribute to shorter hospital stays, lower treatment costs, and better overall outcomes than less modern treatment options. The availability of cutting-edge technologies at private institutions not only enhances clinical results but also significantly improves the quality of life for their patients.^(12)^

This study underscores the systemic barriers that contribute to poor outcomes for Black patients in public hospitals. Delayed diagnostic tests, limited access to specialized treatments, and socioeconomic challenges all contribute to higher metastatic disease rates at diagnosis for patients treated at public hospitals than those treated at private hospitals. Addressing these disparities requires expanding access to early screening programs and improving the availability of advanced therapies in public hospitals,^(12)^ which could help mitigate racial disparities in prostate cancer outcomes.^(13,14)^ Additionally, implementing targeted screening programs focused on Black men could enable earlier diagnosis, reducing disease severity at presentation and alleviating the financial burden on the public healthcare system.^(13)^

The study highlights critical disparities in prostate cancer management in Brazil, addressing a socially and medically important issue. One strength of the study was its robust analysis based on a large and diverse sample of 2,387 patients treated between 2017 and 2024. The inclusion of a public healthcare institution and a private institution ensured a balanced representation of healthcare services in Brazil, allowing for a more comprehensive understanding of disparities. The study also included the same percentage of patients older than 75 years in both groups, providing a consistent basis for evaluating clinical and geriatric decision-making, particularly as survival rates were shown to be worse among this age group. Our findings emphasize that Black (OR=2.27) and mixed-race (OR=1.89) patients are significantly more likely to present with metastatic disease compared to white patients, and those in the SUS are 2.5 times more likely to be diagnosed at advanced stages. This underscores the study's capacity to identify structural inequities, such as geographic barriers, with patients living over 10km from treatment centers being 95% less likely to access robotic surgery (OR=0.05). Furthermore, the study's use of well-defined statistical methods and clear significance thresholds (p<0.01) ensured its analytical rigor. The study's practical recommendations, like targeted screening programs and expanded access to advanced treatments, further highlight its strength in translating data into actionable solutions for policymakers and healthcare providers.

Despite these strengths, the study has several limitations. Only 58% of patients reported their race (1387 patients), with a low proportion of Black (7.35%) and mixed-race (13.84%) patients, limiting the generalizability of racial disparities and introducing a risk of confirmation bias. The analysis of geographic barriers is another area of weakness, as it lacks data on transportation infrastructure or patient-specific locations, which could provide greater context for these findings. A major limitation is that most Black patients were part of the SUS, making comparisons between groups less balanced and caution should be used when interpreting the analysis. The influence of race lost statistical significance in the multivariate model whereas the hospital variable remained significant, suggesting that the disparity is driven more by the healthcare system than by race. Additionally, public healthcare patients were three times more likely to be diagnosed with metastatic prostate cancer, which limited their access to surgery in general, not just robotic surgery. Although the hospitals had high patient volumes, the study's reliance on only two institutions restricts its representativeness across different regions of Brazil, particularly underserved areas. Another limitation was the fact that access to robotic surgery is significantly easier at private services, combined with the fact that most patients in the study were treated in the private system. The lack of robotic surgery for public health system patients and Black patients could be primarily due to the absence of technology at the public hospital, rather than a treatment choice made by healthcare providers. Finally, the lack of detailed socioeconomic data, such as patient income and education levels, limits the depth of the analysis and its ability to fully capture the socioeconomic drivers of the disparities observed.

## CONCLUSION

The findings of this study reinforce the critical role of racial disparities in prostate cancer outcomes in Brazil. Black patients, particularly those treated in the public healthcare system, face significant barriers to early diagnosis and access to advanced treatments, leading to poor outcomes and high healthcare costs. Patients living more than 10 km away from treatment centers also encounter significant barriers to accessing robotic surgery, further amplifying these disparities. Older patients and those diagnosed with metastasis have lower chances of receiving advanced treatments and shorter survival, highlighting the need for targeted interventions for these vulnerable populations. In contrast, private institutions demonstrate better outcomes due to access to advanced technologies and early diagnoses. Public policies aimed at Afro-descendant patients and residents in more distant areas are required to reduce these disparities, improve survival rates, and enhance quality of life. Expanding early screening programs and increasing access to advanced treatments, such as robotic surgery, in public hospitals are essential strategies to mitigate these inequities.

## Data Availability

The underlying data is contained within the manuscript.
